# Supplementary material for: Lacticaseibacillus paracsei HY7207 Alleviates Hepatic Steatosis, Inflammation, and Liver Fibrosis in Mice with Non-Alcoholic Fatty Liver Disease
Source: Int J Mol Sci. 2024 Sep 12;25(18):9870. doi: 10.3390/ijms25189870 (PMC11432063; doi:10.3390/ijms25189870)
Supplement: Supplementary file 1 [file ijms-25-09870-s001.zip › ijms-3189595-supplementary.pdf]

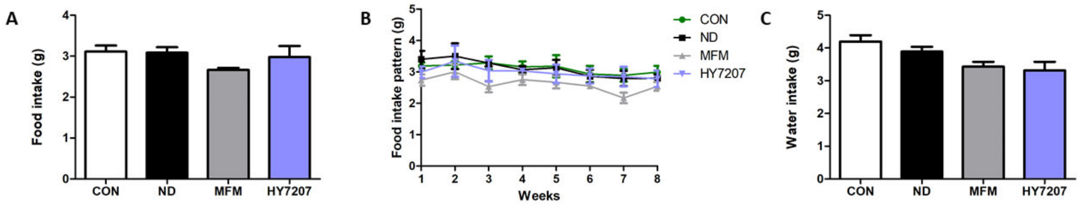

Figure S1. Food intake and water drinking. (A) Amounts of dietary intake (B) Food intake pattern and (C) Water drink during animal experiments. Results are presented as the mean ± SD. CON: Untreated group, ND: NAFLD-inducing diet-fed group, MFM: Metformin group, HY7207: *Lacticaseibacillus paracasei* HY7207 group.

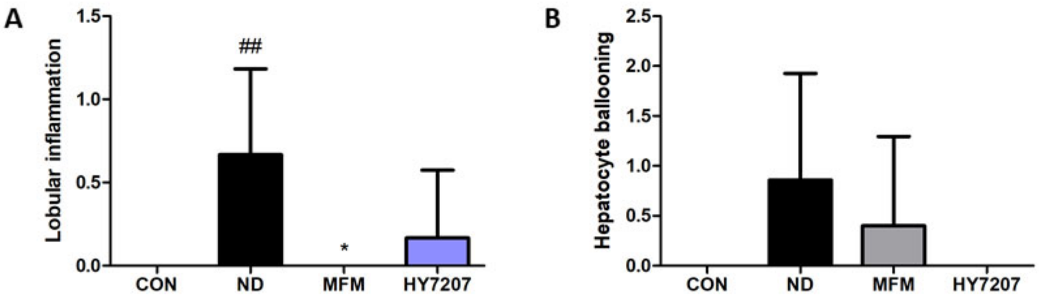

Figure S2. Effect of HY7207 on the hepatic histology of NAFLD-inducing diet-fed mice. (A) Lobular inflammation grade, and (B) Hepatocyte ballooning grade of the mice. Results are presented as the mean ± SD. ## $p < 0.01$ *vs*. the CON group; * $p < 0.05$ *vs*. the ND group. CON: Untreated group, ND: NAFLD-inducing diet-fed group, MFM: Metformin group, HY7207: *Lacticaseibacillus paracasei* HY7207 group.
